# The Family Is My Priority: How Motherhood Frames Participation in Physical Activity in a Group of Mothers Living in a Low Socioeconomic Status Area

**DOI:** 10.3390/ijerph19031071

**Published:** 2022-01-19

**Authors:** Paula Wittels, Tess Kay, Louise Mansfield

**Affiliations:** 1 Division of Sport, Health and Exercise Sciences, Brunel University London, Uxbridge UB8 3PH, UK; louise.mansfield@brunel.ac.uk; 2 Independent Researcher, Leicestershire LE87 2GA, UK; tesskay62@outlook.com

**Keywords:** motherhood, socioeconomic status, physical activity, qualitative, ethic of care, public health guidance, behavior change

## Abstract

Socioeconomic status (SES) is known to influence strongly both life expectancy and healthy life expectancy. Whilst there are multiple factors with complex interactions that provide the explanation for this observation, differences in the uptake of physical activity between high and low SES groups play a role. This in-depth qualitative study set out to understand the response of a group of mothers with young children living in a low SES area of a London (UK) borough to the current physical activity guidance and to investigate whether existing and established interventions based on behavior change are appropriate for this group. A series of three in depth interviews was carried out with the mothers (*n* = 20) over a period of 16 months, and the data collected were analyzed thematically. Four main themes were identified: (1) mothering comes before exercise; (2) mothers are a special case; (3) alone or together; and (4) facilities fail mothers. The mothers were unsure about the benefits of exercise, whether it was relevant for them and how to accommodate exercise alongside their mothering responsibilities. Family and peer group could be both a barrier and a facilitator to participation in physical activity. Without an in depth understanding of the role of physical activity in the lives of mothers of young children, behavior change-based public interventions are likely to fail to meet the needs of this group. A reduction in the current health inequities will only be possible when the needs of the mothers are acknowledged and used as the basis of appropriate public health guidance.

## 1. Introduction

Participation in physical activity is known to play an important role in the reduction in the morbidity and mortality associated with non-communicable diseases (NCDs) including cardiovascular disease, type 2 diabetes, and some forms of cancer [1,2,3,4,5,6,7]. Not all physical activity is equally beneficial; leisure based physical activity appears to be particularly advantageous for health compared to physical activity undertaken as part of a job [8,9]. Socio-economic groups vary in the extent to which they engage in leisure-based physical activity [10,11,12]. Whilst inactivity levels in England are high overall; almost 80% of people fail to meet the recommended guidelines for physical activity, women are less active than men, and low socioeconomic status (SES) groups are less active than high SES groups, even for low-cost activities such as walking [13,14]. These differences in physical activity are apparent in young adults and increase with age [14]. Married women with children are the group least likely to exercise [15,16,17], particularly if their children are under five years old [18].

The explanation for this difference in uptake of leisure-based physical activity is multifactorial. Research with low SES groups has indicated that lack of time plays a role [19,20,21,22] together with a lack of enjoyment of physical activity [20], poor health [15], and being overweight or obese [23]. People who are inactive report that negative emotions driven by poor past experiences or fear, worry, and anxiety about their physical competency are barriers to physical activity [24], and people who are overweight or obese experience stigma trying to exercise leading to exercise avoidance [19,25,26,27,28]. Thus, emotions play an important part and may influence whether inactive or overweight individuals undertake and maintain physical activity.

Understanding the role of physical activity in the lives of mothers of young children is important because of the influence of physical activity on their own health and the way they model and support a physically active lifestyle for their families. Motherhood is complex and diverse, but the dominant understanding, reflected in the literature, is that it represents the ideal state for women and a service to humanity; in contemporary society motherhood is typically associated with selflessness and subordination to men [29]. Although this association reduces when children start school [30], there remains a societal expectation that good mothers give up their own activities so that they can focus on their children [31,32,33]. Motherhood is a diverse experience [34], yet it remains associated with the ethic of care [35] meaning that mothers often measure their worth in the care they provide to others and put the needs of family members above their own [36,37,38,39]. This leads to feelings of guilt if they choose to take time away from their family responsibilities to do something for themselves [38]. Current public health guidance and supporting interventions, many of which focus on achieving individual behavior change, may be ineffectual in this environment if mothers’ focus is not on themselves but on their responsibilities to their families.

Public health interventions are designed to influence individual behavior change and are based on theories including the health belief model [40], the theory of reasoned action [41], the trans theoretical model [42], and the theory of planned behavior [43,44]. Of these, the theory of planned behavior, which takes into account that moving from an intention to change to an actual change is dependent on both the resources open to an individual and the opportunities they have to change, is widely used in health research [45]. The theory has been shown to work well with young, fit, and affluent groups, particularly in the short term [46] but may not serve other groups, such as low SES groups. Other factors outside of the parameters of the theory, not only SES but also environmental factors and health status may influence behavior change [46]. All this serves to question the use of behavior change theory to achieve long term change in low SES groups and emphasizes the need for an in depth understanding of the influences on lifestyle behaviors in specific groups. Only with this understanding can suitable interventions be developed to support the adoption of an active lifestyle [47,48].

Research with low SES groups in England has shown the importance of making public health guidance achievable within the activities of normal daily life, jargon free, and positively framed around capabilities rather than reinforcing a deficit model [49,50]. The infographics published by the UK Chief Medical Officer [51] and intended for use by Health Care Professionals, but actually shared directly with members of the public, have been reported by low SES mothers of young children to be unhelpful and confusing [52]. Instead, such mothers are looking for narratives with which they could identify and achieve [52].

The research reported here investigates motherhood as an influence on participation in physical activity, in a group of mothers of young children living in a low SES area of a London (UK) borough. In order to develop a full understanding of how being a mother suffuses thinking on physical activity and determines response to current guidance an interview strategy was developed which allowed the participants to talk, with confidence and in depth, about the role of physical activity in their lives.

## 2. Materials and Methods

The data reported here were collected from a series of three in-depth interviews with 20 mothers of young children recruited in a low SES area of a London (UK) borough. Whilst SES is associated with health inequalities for women, such findings are inconsistent due to measurement inaccuracies, challenges of data collection, and the dynamic character of SES across the life course [53]. For this research SES was identified in relation to participants’ postcodes which are linked to the UK based Index of Multiple Deprivation [IMD] through Lower-layer Super Output Areas which are small geographical areas of approximately 650 households. The IMD which is divided into 10 deciles measures seven different domains of deprivation: income, employment, health and disability, education and skills, housing, living environment, and crime. All the research participants lived in below average SES areas as measured by the IMD (i.e., in IMD deciles of five or below). The research was approved by the Research Ethics Committee of Brunel University London, and all the participants gave written informed consent prior to the first interview and confirmed their consent at the start of each subsequent interview. Conducting a series of interviews allowed earlier responses to be followed up, with each interview building on the previous one, and responses from other participants could be fed into the discussion [54]. Twenty mothers participated in the first interview, sixteen in the second, and ten in the third. Reasons for dropping out were moving away from the study area, poor physical and mental health, and returning to work. One of the authors (PW) volunteered with the national charity HomeStart, which supports families with young children experiencing difficulties, and established relationships with a SureStart center and children’s centers. The volunteering role involved weekly family visits of two to three hours throughout the research period allowing her to her to meet potential participants and gain their trust for the research [55,56]. The interviews mainly took place in the participants’ homes or at an alternative location selected by the participant and were recorded and later transcribed verbatim by PW. The average duration of the recorded interviews was 48, 52, and 53 min for the first, second, and third interviews, respectively. The shortest interview was 24 min and the longest 72 min. The time spent with the participants was longer than the recorded interview length as time was spent at the beginning and end of each interview that was not recorded. The first interview took place in January 2016 and the last in October 2017.The transcribed interview data ran to more than three hundred thousand words All the interviews were carried out by one author (PW) who was sensitive to the importance of the relationship between the researcher and the participants [57,58,59,60] and saw the data collected as a collaborative knowledge construction [61].

The research methodology combined elements of interpretive and critical theory [62,63,64,65], with a focus on seeing the world through the eyes of the participants and seeking avenues for change. The three interviews each had an interview guide designed to capture the lived experience of the participants and gain an understanding of the of the role of physical activity in that experience. In the first interview, the focus was on the family and household arrangements. The second interview built on this and progressed to the participants’ experiences of motherhood and the interaction between motherhood and their health behaviors. The third interview explored these ideas further and extended the discussion to public health interventions. To ensure validity the research methodology was established at the start of the study, and thick descriptions have been employed to demonstrate the quality of the data interpretation. Data interpretation was also checked through ongoing discussions between the research team.

The participant demographics are provided in Table 1 below. Pseudonyms have been used to protect individual identities and have been selected to be culturally appropriate. All the participants except Yasmine had at least one child under five years old (Yasmine had an eleven-year-old child). The range of ethnicities of the participants reflects the local population [64,65,66,67].

The transcribed data were entered into NVivo v10 (QSR International, Burlington, MA, USA), and the three authors worked together to identify first descriptive categories and then analytical themes and sub-themes. The approach to analysis followed that recommended by Creswell [68] and Miles and Huberman [69], and the principles of reflexive thematic analysis set out by Braun and Clarke [70,71]. The descriptive categories that were considered included the complexities of mothers’ lives, motherhood, mother as an individual, seeking help (from family and peer group), policy, environment, and behavior change. Four main themes were developed: (1) mothering comes before exercise, (2) mothers are a special case, (3) alone or together, and (4) facilities fail mothers. Through the employment of a reflexive approach, together with ongoing review of data and theory, the final themes are an authentic account of the women’s narratives.

## 3. Results

The results are presented by the four main themes with quotes selected from the interview transcripts to illustrate the point being made. The themes explore and describe the role of motherhood in the women’s understanding of their need for physical activity and the benefits it may bring them. The themes also examine the role played by family, access to facilities and opportunities available to the women in the area where they live, on their ability to establish and sustain a physically active lifestyle. The implications of these findings are considered in the “Discussion”.

### 3.1. Mothering Comes before Exercise

The mothers that took part in this study were committed to the ethic of care putting the requirements of their families before their own, with many of the participants describing their needs as being on ’the back burner’.

“Because the family is the first and then you. Especially when you have baby then always the family is number one. You know you can’t just take care of yourself you need to know what the family likes” Fatima

This means that if the mothers are to take part in physical activity, it has to fit around their mothering responsibilities and to a certain extent become part of the mothering role, rather than physical activity being a separate entity. The mothers that undertook physical activity had developed a number of strategies to make this happen. Typically, this meant exercising early in the morning whilst the rest of the family were still asleep or doing something active with the children, which could be walking to school or the shops, dancing with the children at home, or turning a routine activity into an opportunity for physical activity.

“I just walk around the supermarket we go up and down the aisles and I make sure I’m there for about 40 min an hour so I’m getting a walk and he’s getting an outing, you know … it entertains him, builds his social skills, sees other babies and children, other mums and dad so it’s good for him as well.” Sumi

“I try and work my exercise around so it doesn’t have an impact on the house or on my husband. I can imagine it is difficult because I would like to go to the gym yeah I would like to go and join a health you know club and do some sort of swimming you know but it is what it is” Kirti

Nevertheless, for the study participants, the mothering role retained its primacy, and for many of the mothers, this meant that their physical activity was restricted by the changing needs of their children. Women that had been able to walk briskly pushing a buggy found that they could now only walk short distances and more slowly when their children outgrew the buggy. The mother’s physical activity stopped during the school holidays, and some women were no longer able to exercise at home when their children’s paraphernalia filled the space at home. Mothers also gave up their opportunity to exercise to look after other family members who needed support.

“he [her husband] also bought me a wii fit board a few years ago but again we haven’t got the space for me, but I was using that and I was finding that really helpful…I was doing a lot of running using the um wii fit cause I just had the strap on my arm so that was really helpful but we just don’t have the space to put that out just at the moment” Shabnam

The mothers’ ability to exercise was made all the harder because of the lack of support for their mothering activities from other family members. In this group of mothers, there was very little in the way of sharing the caring responsibilities. Many of the partners worked long hours and/or night shifts and were not available or willing to look after the children so that the mother could exercise. For example, Meenakshi explaining why she could not go swimming:

“I mean he would [look after his daughter] but he would only give me half an hour so if I wanted to go it would be like OK come back by seven o’clock and it’s already six o’clock and if I take the time to drive there change …he says I’ve got other things to do in the house cooking and cleaning and tidying up. He doesn’t think that I should have time to myself kind of” Meenakshi

An interconnected way of expressing the priority put on looking after the family was revealed in discussions about the affordability of taking part in physical activity. From the mothers’ point of view, the cost of physical activity such as gym membership or exercise classes, together with the cost of accessing these facilities and the cost of childcare, was for many, an inappropriate use of family funds. They did not want to spend money on themselves that could be used for the benefit for the family. For example, Alison talking about the cost of physical activity at the local leisure center:

“just prohibitive … I just think what they offer for cost is far too much, it’s far too much for us to, for us to contemplate” Alison

The clearest manifestation of mothering taking precedence over exercise was in the mothers that had been physically active before they became mothers but had given up on becoming a mother and remembered it wistfully and hoped that they would be able to return to it at some point in the future when their mothering responsibilities allowed. Not all the women had exercised before becoming mothers and had therefore not all had experienced this loss.

“before I got married I was going for kick boxing……I was happy there and sometimes I was also running and I was working and sometimes I had to run for five kilometres so I was also running, now I’m not doing anything……I think it’s because I’ve got children, that’s why I don’t have time and don’t have I can’t go out and do exercise because it’s just changing my life……” Naseem

“I miss being fit, I miss being active I miss my usual fitness” Lilly

### 3.2. Mothers Are a Special Case

The women that participated in the research were divided on whether they thought participating in physical activity was appropriate for mothers of young children. Those that deemed it inappropriate gave two main reasons for their view that mothers were a special case, exempt from the guidance on physical activity; one was that the bodily changes that they had undergone during pregnancy and giving birth had led to it being too difficult for them to exercise, and the second was that they were active anyway and therefore had no need for further exercise. These two contrasting participant views are illustrated in the following two quotes:

“I’ve been in so much pain…I can’t do exercise, physically can’t. Walking is the only thing I can do” Jade

“Oh my goodness I do the housework. I think that’s enough exercise. And that keeps me fit as well” Nori

The mothers who felt physical activity was not something they should be doing talked about the physical changes they had experienced and how exercise caused them pain and discomfort. Their change in body shape made them feel awkward and out of place in formal exercise settings. This feeling was accentuated because they could recall their pre-pregnancy selves which would have in their eyes been accepted in such places. Their belief that exercise was not something they should be doing was supported by the unsuitability, in their view, of the type of exercise that was offered, too strenuous for mothers of young children. Another aspect of this viewpoint was a fear of failure, especially for those mothers who had been active in the past; they thought that their new bodies might let them down. They saw participation in physical activity as having a lifecycle, and as mothers, they were in a part of the cycle where physical activity was not required.

“A lot of women don’t exercise at this time…The confidence in myself because now I don’t have a lot of confidence. You know when your body change you don’t have this confidence in yourself” Fatima

“You’ve got be careful, you mustn’t do that, you can cause yourself an injury…I suppose at the end of the day I listened to like the negativity like the negative comments” Shabnam

“I haven’t thought about that [exercise] yet…and then exercise probably in a few years’ time when the children are a bit older then it will follow along” Christine

The mothers who believed that they had no need for further physical activity referred to how busy they were with childcare and housework. They had no interest in relating their activity to current physical activity guidance, believing such guidance was irrelevant to them as busy mothers.

Other mothers in the group had a different perspective. They were committed to exercising, but this was not something they did for themselves. Again, they saw themselves as a special case, but their view was that mothers need to exercise to ensure that they were sufficiently healthy to look after their family. Exercise was not something they did for themselves but rather something they did as part of their mothering role.

“I think we do it more because we’ve got young kids we need to be running around and be healthy more for that. If that makes any sense so that we’ve got stronger bodies be able to you know take the kids to the park go for walks and be active with them. As opposed to do you know what I want to lose weight and look good and want to get toned” Kirti

Related to this idea that physical activity was something mothers did for their family, rather than for themselves, was that some of the mothers thought they could serve as a role model for physical activity for their children by providing physical activity for their children without actually participating themselves. Several of the mothers for example talked about swimming with their children, but what they were actually doing was standing in the pool supporting their children, whilst their children enjoyed the water. Others sat and watched whilst their children took part in gym and dance classes.

### 3.3. Alone or Together

The mothers’ responses on family influences and social connections were analyzed under this theme. Some of the mothers that participated in the research came from a family with a culture of exercise and were regular exercisers before they became mothers. These mothers saw their family culture as supportive and felt that the time would come when they would be able to exercise again, even if current mothering responsibilities were preventing them from doing so. These mothers felt that they could ask their partners or other family members for support with childcare so that they had time to exercise. None of the mothers talked about going outside the family circle for this type of support. Friends, however, were seen as possible sources of motivation to start or maintain an exercise program, but they were not seen as a childcare resource. Gym discounts for friends that joined together were taken up by some of the mothers in the study, a clear benefit of cooperation. Virtual friends could also serve as a source of inspiration and motivation for physical activity. In contrast, the mothers that did not have a family culture of physical activity felt isolated with no encouragement to participate in physical activity.

“initially three of us joined together so we got discount. It was much cheaper because three of us joined. But individual fees is much higher, compared to others…we are continuing together, we go together… I don’t think I would go alone …it’s like motivation kind of let’s go don’t be lazy let’s go together” Aahna

“Motivation is a problem sometimes but then I keep motivating myself ah. There are Facebook groups of like healthy eating and exercises group. I’ve joined that and I keep reading that when I feel depressed or when I feel demotivated I keep reading that and then I get motivated to exercise. People are so busy and they are exercising then I can do it.” Rozina

For the families with a culture of physical activity that saw participation in physical activity as part of their family life, there was a desire to make physical activity a family event rather than something the mother did on her own. In most cases, the mothers were expressing ideas for the future rather than their current reality because their children needed to be older for the activities they envisaged. As a second best or short-term option, they would have liked the opportunity for their children to do an activity whilst they exercised close by, but unfortunately this facility was not offered at the local leisure centers.

“we’ve already said we’ll all get bikes and stuff, go out bike riding, and walking and just do lots of you know just open it up for him you know, give him a chance, get him a bike, go out bike riding, get him a kite, go kite flying” Sumi

Input from peers was not always positive. In some cases, friends could serve as a barrier to participation in physical activity by doubting the mothers’ capability to exercise, suggesting that she could harm herself through exercising.

### 3.4. Facilities Fail Mothers

Facilities is used here in a broad context embracing both the actual offering of physical activity but also the accessibility of what was offered and whether the mothers participating in the study had the opportunity to access physical activity. A good illustration is that some of the mothers referred to the availability of free exercise equipment in the local parks and spoke positively about it, but none of them had used it. Many of the mothers complained about a lack of information on what was available locally to support them with physical activity. As set out above they felt that even if exercise classes were made available, they were not tailored to their needs. A lot of what was provided was seen as short term and unreliable whilst what they were looking for was a reliable long-term solution. Accessing facilities in the dark, in what were considered unsafe streets, was another problem as was the need for female only sessions. The lack of physical resources drove some of the mothers to Facebook groups which they saw as a good source of information and support.

“And so they do have stuff but it’s dotted all around like far away from where I can get. Not a practical option at all” Vicki

“I’m finding is everything is very focused on newer mums not me with her who is now three” Alison

Whilst some of the mothers said they would like to participate in physical activity, there was also a concern expressed that they might fail which prevented them from starting. The opportunity needed to be provided in a way that made it easy to participate to encourage regular attendance and allow them to build up their activity:

“I’d also like to just try something that’s regular up the road that’s not going to cost me anything and see if I can stick to that. Start smaller … and hopefully actually stick to a proper kind of routine with exercise because that’s never been part of my daily routine” Vicki

Vicki who said “I take no exercise” was actively looking for local opportunities for physical activity and had found out about a local program that allowed access to a range of sport sessions, but she was not able to access it as the referral she needed from her General Practitioner was not made despite her requesting one.

The cost of exercise was a barrier for many of the mothers and some gave as an explanation that they had recently moved from having two earners in the family to one. In the context of reduced income and time pressures, they found the requirement to commit to regular expenditure daunting. Whilst some facilities offered a pay as you go arrangement, this could mean a reduced offering such as no access to the crèche, which does not represent good value for money for mothers.

“Too much everything wants you to sign up to direct debits for this that and the other um you know committing yourselves weekly or monthly and we just can’t do that at the moment you know” Alison

To encourage participation, promotion of physical activity and facilities has to fit the mothers’ image of themselves. Messages that do not fit how they see themselves are likely to be ignored. For example, this is Christine talking about an image from the “This Girl Can” campaign, a national lottery funded Sport England project, designed to encourage women of all shapes, sizes and fitness levels to exercise:

“this girl can it’s for mother and daughter and the daughter is a teenager but my daughter is seven years old so I didn’t really go deeper” Christine

The mothers had an alternative vision for the type of facility they were seeking. As well as addressing their concerns with current provision which meant better information, more diverse provision for all mothers and not just new mothers, and reliability in delivery, they articulated the need for a self-help group comprising local mothers where they could share experiences and support each other.

“somebody who can guide me, talk to me, answer questions motivate me you know, same might be with other mums as well or other people” Kirti

The mothers that participated in the study were seeking a service designed to meet their specific needs:

“I’m saying you know they should have something groups which are people with like-minded in a sense, mums to go out to have a session” Lilly

## 4. Discussion

The data analyzed in the four themes demonstrate the complexity of the women’s lives and sheds light on why public health interventions that focus on the individual in isolation from her mothering responsibilities and living environment are unlikely to bring about behavior change. Mothers from low SES groups represent an important target for public health interventions seeking to increase the uptake of physical activity. Local coordination across the health and leisure sectors in physical activity provision is often missing. The results reported here indicate that a detailed understanding of how motherhood influences attitudes to physical activity is essential for the development of appropriate guidance and interventions. The mothers in this study see themselves as having distinct needs when it comes of physical activity provision and participation and are not convinced that the current public health guidance applies to them. Without targeted public health interventions to support the uptake of guidance in this group, health inequalities are likely to persist and may even increase as non-targeted interventions are disproportionally taken up by higher SES groups [72,73] thus widening the gap between high and low SES groups.

Public health interventions are typically based on behavior change theory [74] despite its acknowledged shortcomings in achieving long term behavior change in low SES groups [46]. The results reported here provide a clear perspective as to how guidance needs to be framed to be relevant to mothers of young children living in a low SES area and to increase the likelihood that women will become more active within the parameters of motherhood. Firstly, the guidance and related interventions must support the women in their mothering role rather than seek to take the mothers away from their children. Conflict between mothering and physical activity needs to be avoided. Secondly, the women should be encouraged to see physical activity as something they are doing for both their own enjoyment and health as well as to support the family. This is a more positive framing than the concept of the “third shift” described by Dworkin and Wachs (p610), [75] where physical activity becomes another task for women to complete after their paid work and unpaid work in the home. The benefits of positive framing or “gain framing”, particularly the social and mental health benefits that will accrue from participation in physical activity have been highlighted by others working with low SES groups [49,50,52].

Some mothers will require more encouragement and support than is currently available to them to become physically active because they are starting from a position that denies the need to undertake exercise, either because they feel physically unable to exercise or because they feel their everyday activities provide sufficient exercise. To change, mothers will need information presented in a way that they can readily adopt it in their busy lives, acknowledging both social constraints and inherent anxieties. Salmon et al. [52] found that mothers responded positively to “like me” stories with which they could identify. The mothers that participated in this study are part of a general trend in the developed world away from an active lifestyle towards increased sedentary behavior [3]. Much of their work outside the home was sedentary in nature and leisure was typically screen based. Whilst some of the women walked to access facilities, some had the use of cars. Physical activity can mitigate the adverse impact on health of a sedentary lifestyle [1,76,77], but the non-exercising mothers in the study were not aware of its necessity. Establishing the need to exercise will not be sufficient for those women that feel that they are physically unable to exercise. Poor physical and mental health can serve to prevent participation in physical activity [19,20,21]. There is a high level of morbidity in low SES groups [19,78,79], which suggests that achievement of behavior change in this group will require a more holistic approach to the health of the individual.

The results reported here show that whilst it is essential to understand the perspective of the mothers, the mothers are not acting in isolation in deciding whether or not to participate in physical activity. They are negotiating complex demands of motherhood that arise from individual, family, and societal expectations that shape their values and hopes, which in turn shape their behavior. The women who took part in the study undertook a disproportionate part of the childcare and housework responsibilities compared to their male partners. This is in line with a recent study of the division of household work in the UK which found that the gendered division of housework and childcare was most traditional when women are not in paid work or are in part time work and have children living at home [80]. Some of the participants with supportive families were able to negotiate time away from their mothering responsibilities to exercise whilst others could not. Women in low SES groups typically find such negotiations more difficult [78,81,82]. In these difficult circumstances, peer group support can make it easier for women to undertake and maintain an exercise regime [56,83,84].

Even if women are committed to making physical activity part of their lives, the results reported here make it clear that they are not adequately supported in the environment where they live. Some of the mothers had enterprising solutions to the difficulties in walking in unsafe streets in poor weather, such as making shopping in the local supermarket both a walk and an outing, but generally, they were disappointed by what was on offer in their local community. Facilities were either physically out of reach or considered too expensive for families with limited means. This finding echoes that of Jones, Hillsdon, and Coombes, [85] in England (Bristol) who found that even when physical activity facilities and green spaces were available in poor areas, they were not used as well as similar facilities in more affluent areas. Moreover, some of the women feared the reaction of others to their changed bodies and gave that as an explanation for not exercising. Women that consider themselves out of shape feel stigmatized and unwelcome in exercise venues [19,26] and this can lead to exercise avoidance [27].

All the women that participated in the study had motherhood in common as well as living in low SES neighborhoods. They were however diverse in terms of their ethnic background. We were not able to comment on the impact of ethnicity on our findings, but this would be an interesting area for further research.

## 5. Conclusions

Mothers of young children living in low SES areas are a key target for physical activity guidance and interventions. Current behavior-change-based programs with a strong focus on the individual fail to meet the needs of this group. This in-depth qualitative exploration of the lives of the mothers demonstrates that the mothers have particular priorities and needs that influence their desire and ability to participate in physical activity. Unless these are acknowledged and addressed, behavior change in this group is unlikely. Specifically, physical activity needs to be framed as compatible with the mothering role and made available in a format that the mothers can access. Some women may require additional support in terms of their overall health, negotiation skills, and body confidence before they are ready to embark on physical activity and reassurance that being active is compatible with good motherhood.

## Figures and Tables

**Table 1:** Participant demographics.

Interviewee (Number of Interviews)	Age	Ethnicity	No of Children	Living with Partner
Sumi (2)	39	Indian British	1	yes
Fatima (3)	25	North African	1	yes
Naseem (3)	40	Pakistani Danish	3	yes
Vicki (2)	32	White South African	1	yes
Radhika (2)	31	Indian	1	yes
Aahna (1)	29	Indian	1	yes
Eva (2)	31	White Eastern European	1	yes
Christine (2)	42	Filipino	2	yes
Jade (3)	37	Indian British	2	yes
Rozina (1)	32	Indian	1	yes
Shabnam (3)	44	Pakistani British	2	yes
Eisha (3)	24	North African	1	yes
Nori (3)	29	Pakistani British	3	yes
Kirti (3)	37	Indian British	3	yes
Lilly (1)	34	Sri Lankan British	1	yes
Meenakshi (3)	32	Indian British	1	yes
Rachel (3)	41	White British	1	no
Jaswinder (2)	29	Indian British	1	yes
Alison (3)	43	White British	1	yes
Yasmine (1)	43	Pakistani British	1	yes

## Data Availability

The data that support the findings of this study are available in the PhD thesis Wittels PY Shaping health: understanding and influencing lifestyle behaviors in low socioeconomic women, Brunel University, London, 2021.
